# Morphological control of cuprate superconductors using sea sponges as templates†

**DOI:** 10.1039/d5ra00541h

**Published:** 2025-04-17

**Authors:** Jan Maurycy Uszko, Jula C. Schroeder, Stephen J. Eichhorn, Avinash J. Patil, Simon R. Hall

**Affiliations:** a The Bristol Composites Institute (BCI), School of Civil, Aerospace and Design Engineering, University of Bristol, University Walk Bristol BS8 1TR UK; b School of Chemistry, University of Bristol Bristol BS8 1TS UK simon.hall@bristol.ac.uk

## Abstract

Functional porous superconducting sponges, consisting of YBa_2_Cu_3_O_6+*δ*_ (YBCO) and Bi_2_Sr_2_CaCu_2_O_8+*δ*_ (BSCCO), were created by biotemplating with natural sea sponges. Naturally occurring calcium in the spongin fibers was utilized to dope YBCO and to form BSCCO without adding any external calcium source. The sample morphology was confirmed with scanning electron microscopy, and the sample composition was confirmed with energy-dispersive X-ray spectroscopy, powder electron diffraction and high-resolution transmission electron microscopy. The YBCO sponge exhibited a critical temperature (*T*_c_) of approximately 70 K, and the BSCCO sponge showed a *T*_c_ of 77 K. This proof-of-concept study demonstrates the feasibility of using sea sponges as a greener, more sustainable template for superconductor synthesis.

## Abstract 2

Functional porous superconducting sponges, consisting of YBa_2_Cu_3_O_6+*δ*_ (YBCO) and Bi_2_Sr_2_CaCu_2_O_8+*δ*_ (BSCCO), were created by biotemplating with natural sea sponges.

## Graphical abstract

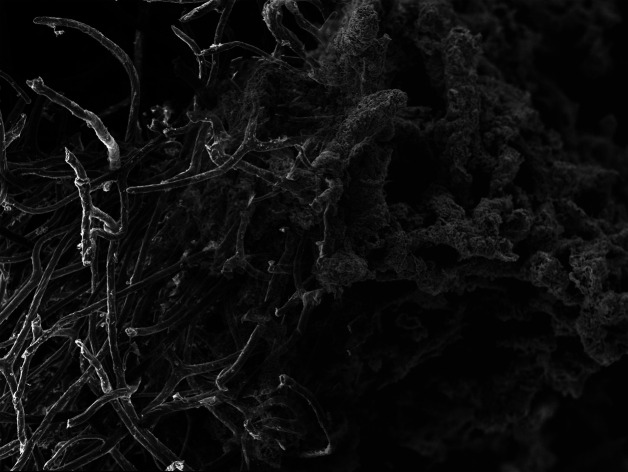

## 1 Introduction

The synthesis of complex functional materials remains a central focus in materials science for advancing the quality and targeted morphology of superconductors. Template-assisted syntheses have proven invaluable for creating nanostructured superconductors with enhanced properties.^1,2^ For instance YBa_2_Cu_3_O_6+*δ*_ (YBCO) and Bi_2_Sr_2_CaCu_2_O_8+*δ*_ (BSCCO) sponges have been synthesised using melamine-formaldehyde foams, commercially known as “Magic Eraser”.^3^ Despite the effectiveness of such templates, their environmental impact and other limitations underscore the need for sustainable, biobased alternatives.

Examples of biobased templates, including pollen and cuttlebone, offer a promising route for sustainable material synthesis.^4,5^ Similar to synthetic templates, naturally sourced biopolymers can act as chelating agents in the production of metal oxides and provide nucleation sites within a matrix, influencing crystal morphology.^6^ This often leads to materials adopting intricate 3D structures that replicate the original architectures of the biopolymer even after calcination.^1^ Nature's evolution has meticulously perfected biobased templates featuring complex architectures and chemistries. These have increasingly become a focus of research, highlighting their potential in advanced material synthesis and sustainable technologies.

Sponges (*Demospongiae*) have established themselves as a natural renewable source of spongin.^7,8^ This microfibrous structural biocomposite possesses complex chemistry and intrinsic 3D architecture which is similar to the artificially designed biobased porous organic materials which have been previously used in the synthesis of superconductors *i.e.* chitosan and dextran.^1,9^

The exact composition of spongin remains unknown; however, its primary structure is proteinaceous, with notable concentrations of calcium and silica.^7^ This composition makes spongin an ideal template for the BSCCO system, where calcium is a critical component. In addition, the unique porous and renewable spongin template may produce lighter and more efficient functional materials in applications where weight is of critical importance.

Sea sponge farming can be easily done with minimal processing and transport costs, making it ideal for developing coastal communities.^10^ It is known to provide a sustainable place of employment for women in Zanzibar, through non-profit organisations like Marine Cultures.^11^ Besides their uses in beauty products sea sponges are farmed and harvested to extract metabolites.^10^ This ensures that there are existing supply chains for sponges to be used as templates in chemical syntheses. BSCCO is the first superconductor with a *T*_c_ over the boiling point of liquid nitrogen that doesn't contain any rare earth metals,^12^ making it more sustainable compared to other superconductors. A combination of those two factors makes BSCCO templated sponges extremely appealing as an environmentally friendly alternative to conventional superconductor synthesis. This approach also aligns with multiple Sustainable Development Goals (SDGs), including no poverty (SDG 1), gender equality (SDG 5), clean water and sanitation (SDG 6), decent work and economic growth (SDG 8), sustainable cities and communities (SDG 11), responsible consumption and production (SDG 12), and life below water (SDG 14).^13^ This combination of environmental sustainability and socioeconomic benefits highlights its potential as a model for sustainable development and emphasises the importance of this proof-of-concept study.

In this paper, we demonstrate the feasibility of using horny sponges as a template for the synthesis of cuprate superconductors, improving on previous methods by employing a natural, sustainable material with an intrinsic 3D architecture. This strategy allows us to explore whether the sponge's inherent calcium can be directly integrated into the superconductor structure while replicating the sponge's porous morphology in the final material.

## 2 Results

### 2.1 Sponge

**Fig. 1:**
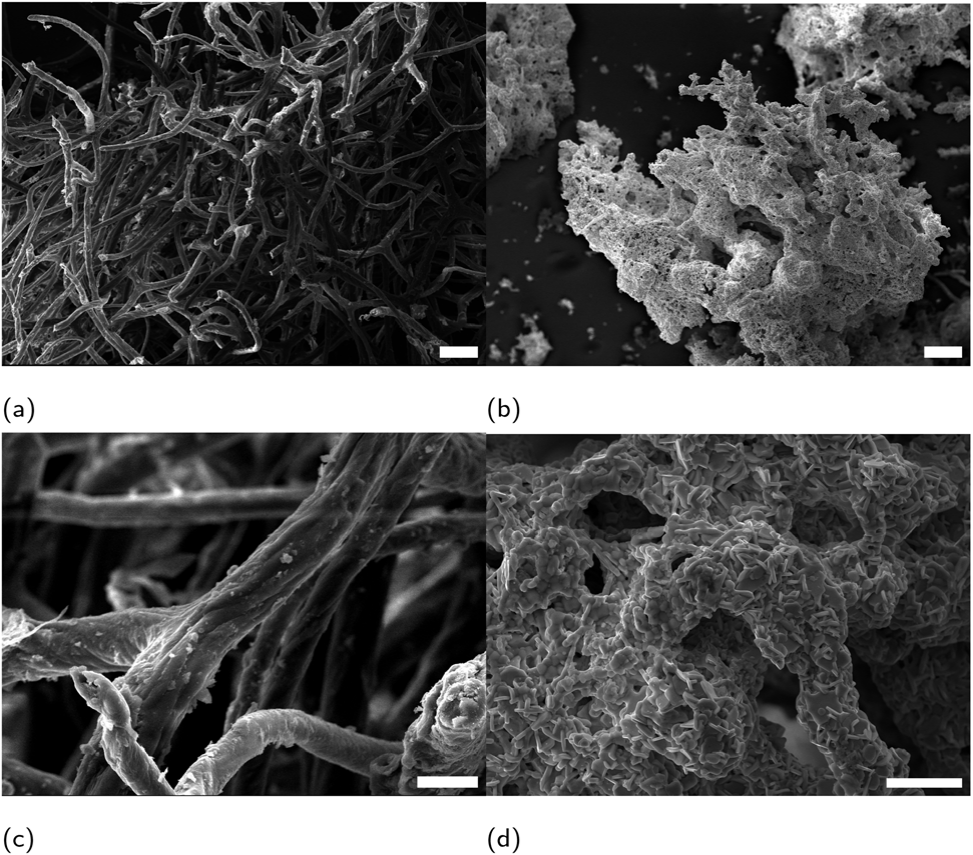
SEM micrographs showing (a) and (c) untreated, (b) and (d) calcined sponge. Scale bars: 100 μm in (a) and (b) and 20 μm in (c) and (d).

**Fig. 2:**
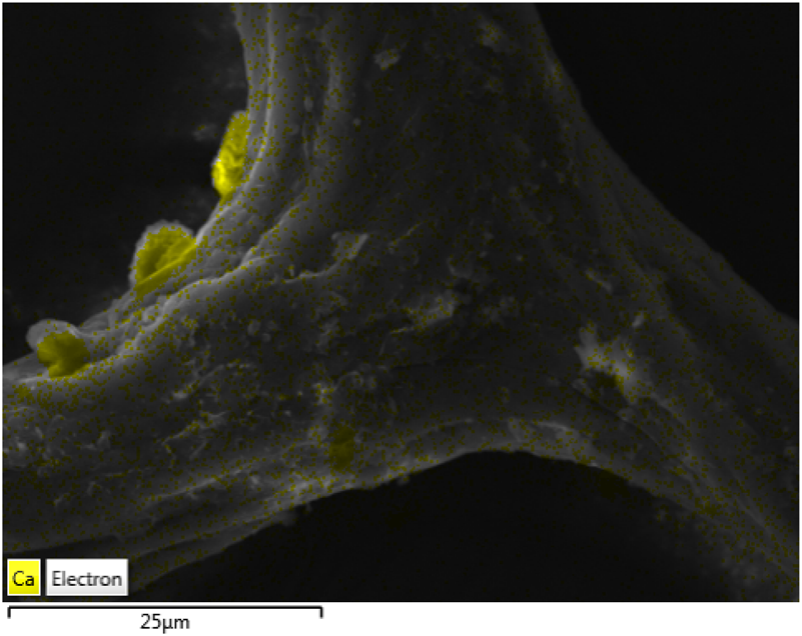
EDX map of a spongin fibre. The calcium is highlighted in yellow.

Scanning electron microscope (SEM) micrographs in Fig. 1 show the characteristic morphology of an untreated demosponge sponge (Fig. 1a and c) with long intertwined spongin fibres.^14^ After calcination (Fig. 1b and d), the overall porous architecture is retained (visible even in higher-magnification images), although some fine features of individual fibres are lost. The preserved porosity could enable more efficient cooling of a superconducting material by preventing the formation of local hotspots.^15^ An energy-dispersive X-ray (EDX) elemental map in Fig. 2 identified discrete calcium rich regions on spongin fibres. Although keratose demosponges themselves do not produce calcitic spicules, they often grow on calcareous substrates, which may account for the presence of calcium incorporated in the spongin structure.

### 2.2 YBCO

**Fig. 3:**
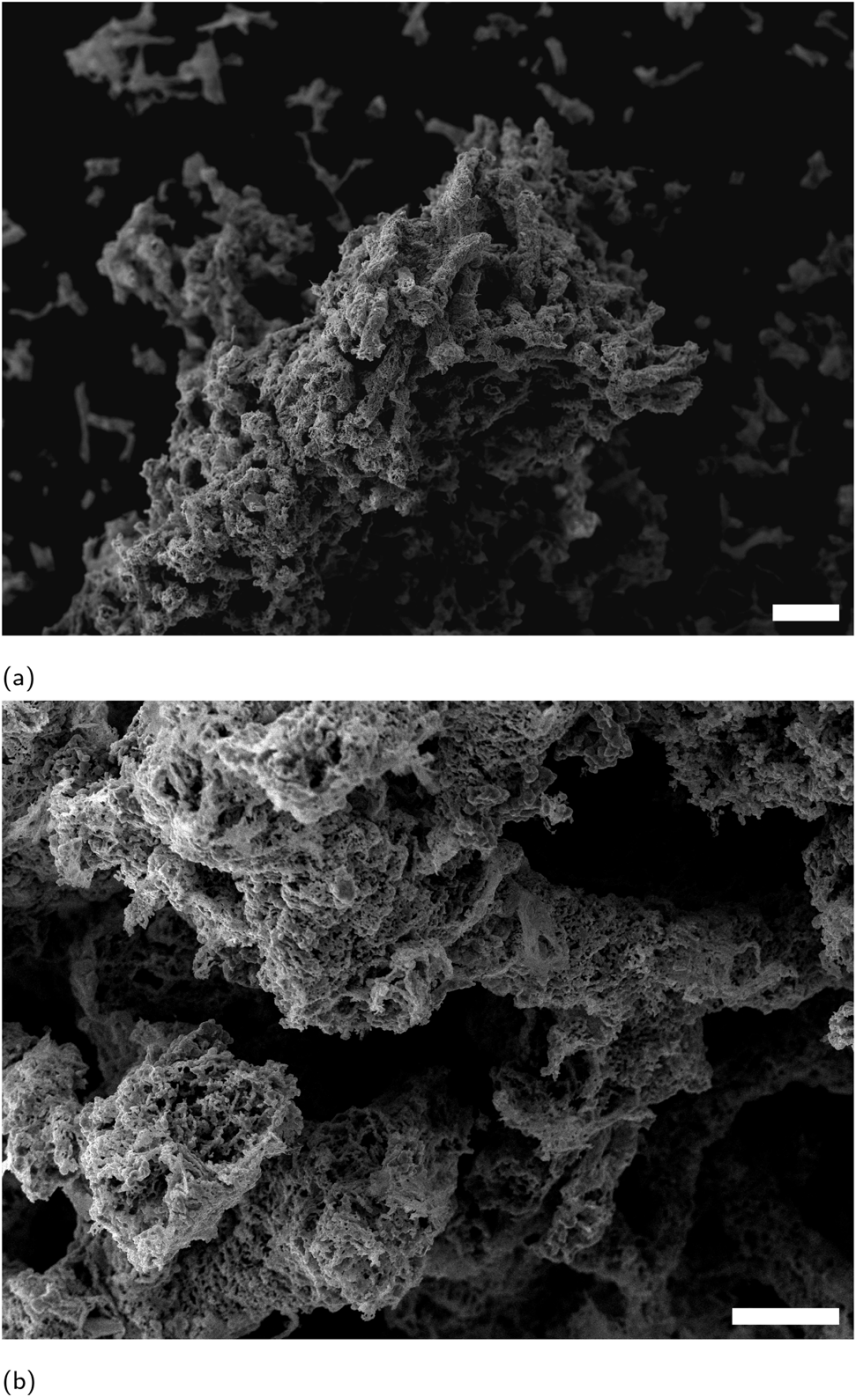
SEM micrographs showing YBCO sponge. Scale bars are 100 μm in (a) and 20 μm in (b).

**Fig. 4:**
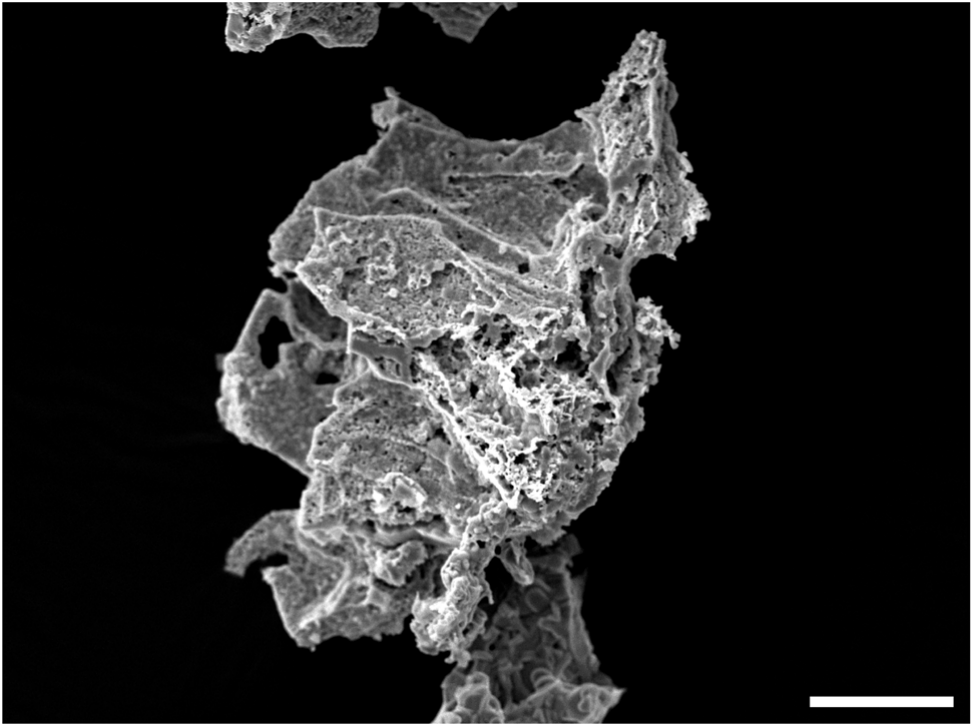
SEM micrograph showing YBCO prepared without the use of templates. The scale bar is 20 μm.

**Fig. 5:**
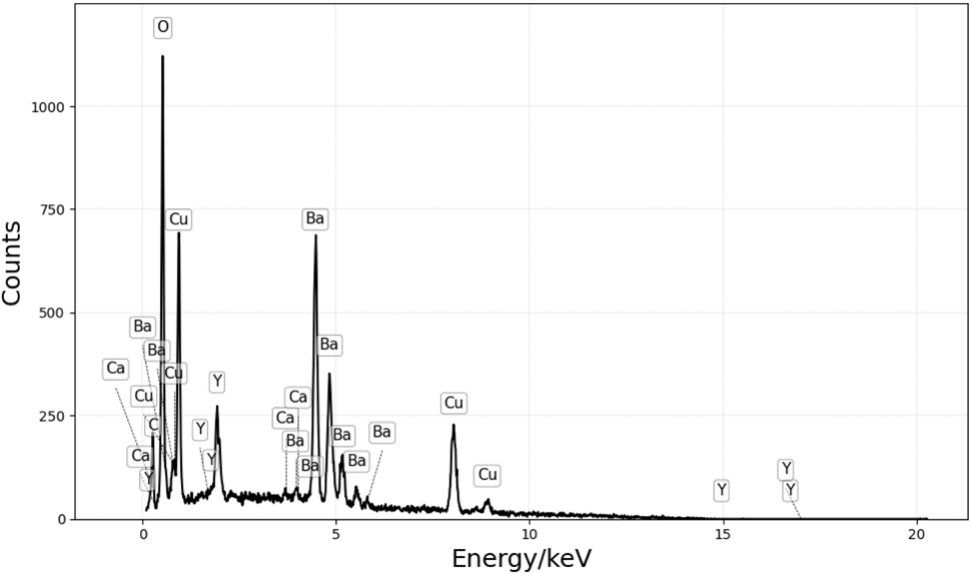
EDX spectrum of YBCO sponge.

The YBCO sponge has been prepared as described in the ESI.† However, briefly, sea sponges were infused with a precursor solution and calcined at a specific temperature. SEM micrographs of the YBCO sponge in Fig. 3 show that the porous structure of the calcined sponge is largely preserved in the treated specimen, and lower magnification images highlight long fibre-like strands are evident, reflecting the template morphology; in contrast, these fibrous structures are absent in a control sample prepared without any template (Fig. 4). EDX analysis in Fig. 5 confirmed the expected elemental composition of YBCO (yttrium, barium, copper, oxygen) and also detected calcium originating from the sponge template.

**Fig. 6:**
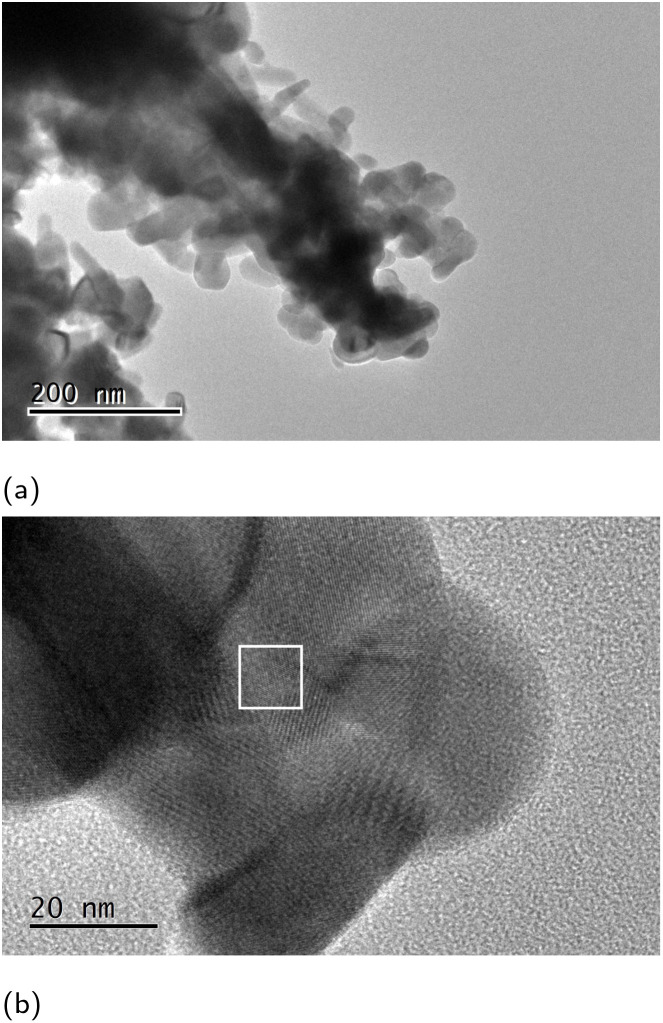
High-resolution TEM micrographs showing the sub-structure of the YBCO sponge. The scale bar in (a) is 200 μm and 20 μm in (b). The *d*-spacing measured in the white box in image b 1.57 Å corresponds to a crystallographic (213) plane indexed according to the Inorganic Crystal Structure Database reference #65549.^16^

High-resolution transmission electron microscope (HR-TEM) micrographs of the sponge in Fig. 6 show a distribution of small highly crystalline particles (Fig. 6a), with characteristic lattice and Moiré fringes seen in Fig. 6b. The presence of the YBCO is confirmed by the indexing of the *d*-spacing of the lattice fringes in Fig. 6b.

**Fig. 7:**
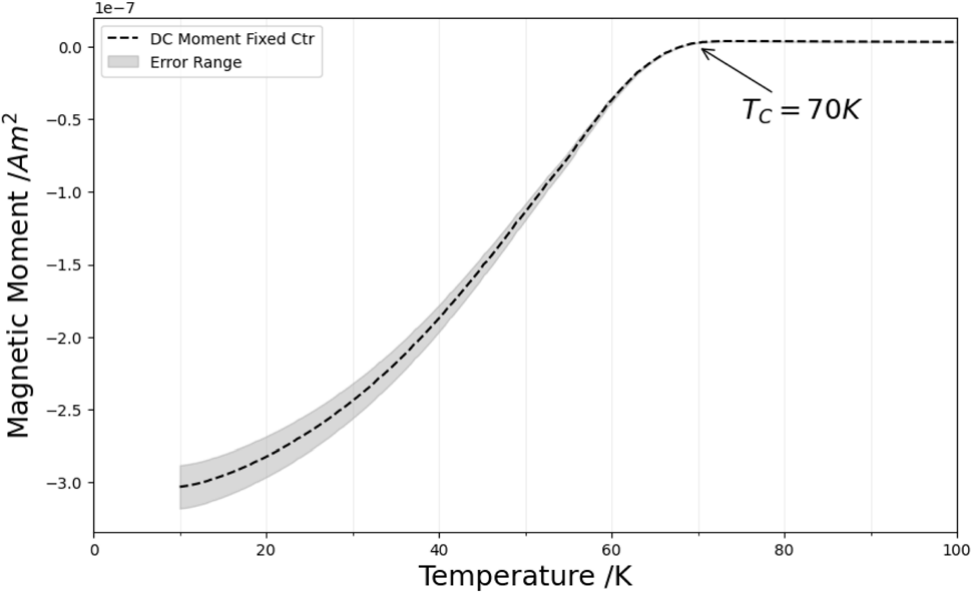
SQUID magnetometry measurement of YBCO and BSCCO.

Further confirmation of the indexing as YBCO was achieved *via* Rietveld refinement of the powder X-ray diffraction (PXRD) pattern in Fig. S1.† Refinement identified the presence of superconducting phases, 38% of which was identified as calcium-doped YBCO, with a *T*_c_ between 77 and 83 K.^17^ Superconducting quantum interference device (SQUID) magnetometry measurements in Fig. 7 confirmed the superconductive properties of the material. The *T*_c_ was, however lower than expected, possibly due to the formation of small crystallites of YBCO, which have been reported to have a *T*_c_ lower than that of bulk YBCO.^18^

While the superconductivity of the YBCO sponge has been reduced, the ease with which calcium from the spongin fibres was incorporated into YBCO highlights the potential of this novel biotemplate for doping superconductors without additional external calcium sources. This outcome naturally led to the investigation of BSCCO, a well-known high-temperature cuprate and calcium-containing superconductor. Its composition and structural characteristics make it an ideal system to further exploit the unique advantages offered by the proposed biobased template approach.

### 2.3 BSCCO

**Fig. 8:**
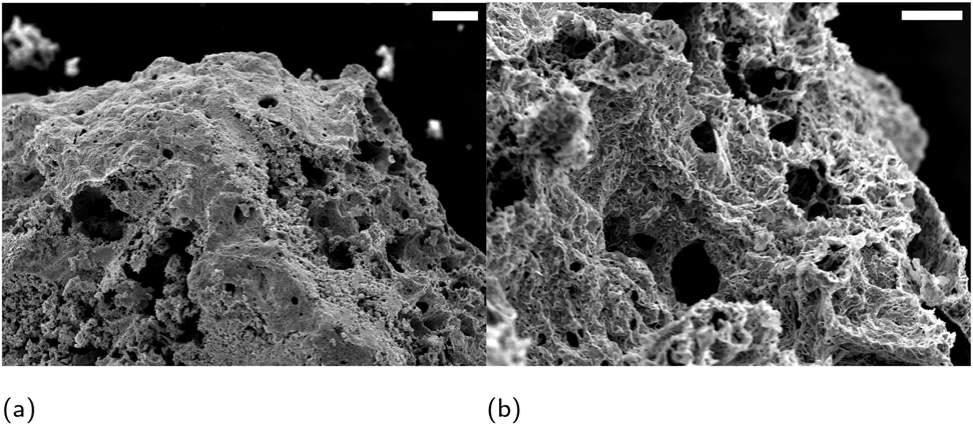
SEM micrographs showing BSCCO sponges. Scale bar in (a) is 100 μm and 20 μm in (b).

Encouraged by the results with YBCO, we next prepared a BSCCO sponge in a similar manner (see ESI for details†). The BSCCO-infused sponge retained the characteristic porous structure of the template after calcination. Fig. 8.

**Fig. 9:**
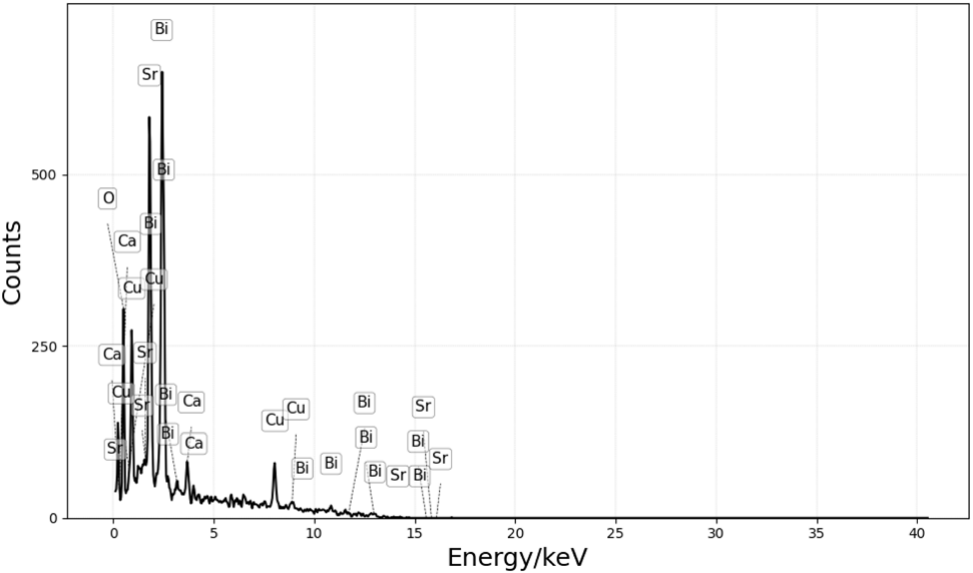
EDX spectrum of a BSCCO sponge.

Based on the YBCO findings, the BSCCO precursor solution was deliberately formulated without calcium, to test whether the sponge's inherent Ca could suffice. Indeed, EDX analysis of the calcined sample, in Fig. 9, confirmed the presence of bismuth, strontium, copper, calcium and oxygen.

**Fig. 10:**
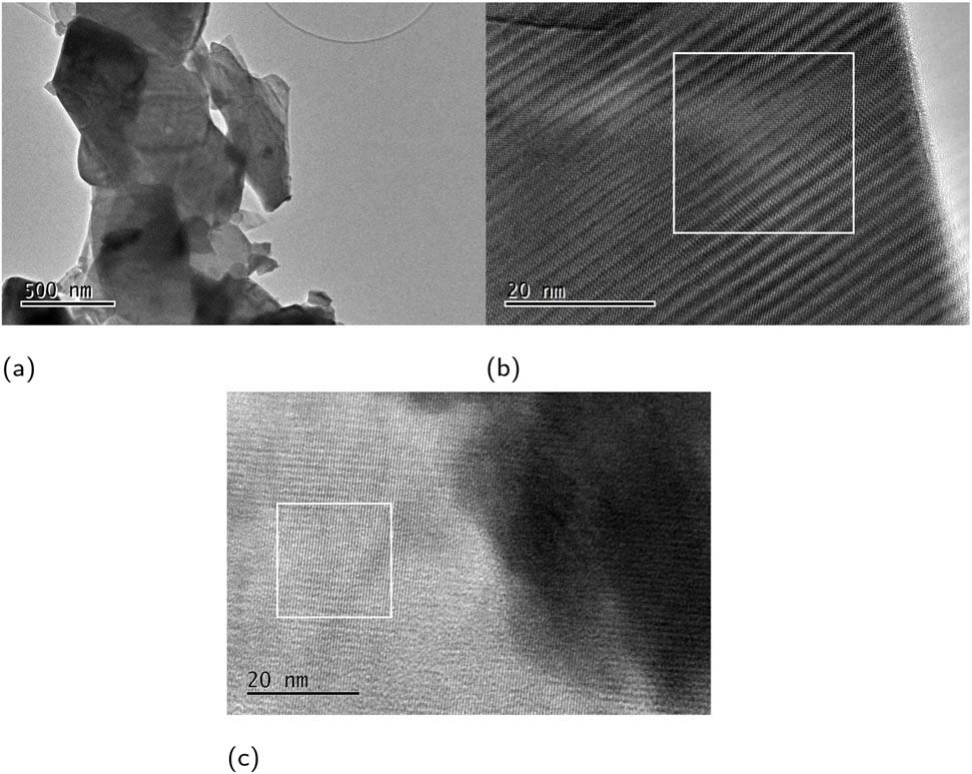
TEM micrographs showing a BSCCO sponge. Scale bar in (a) is 500 μm and 20 μm in (b) and (c). The *d*-spacing measured in the white boxes in (b) is 2.71 Å corresponding to the 
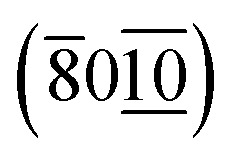
 plane and 5.32 Å corresponding to plane (1̄11) in (c). Indexed according to the Inorganic Crystal Structure Database reference #203210.^19^

HR-TEM micrographs of the BSCCO sponge in Fig. 10 show a distribution of highly crystalline particles (Fig. 10a).

The structure of the crystallites has been confirmed as BSCCO by the indexing of lattice and Moiré fringes in Fig. 10b and c.

**Fig. 11:**
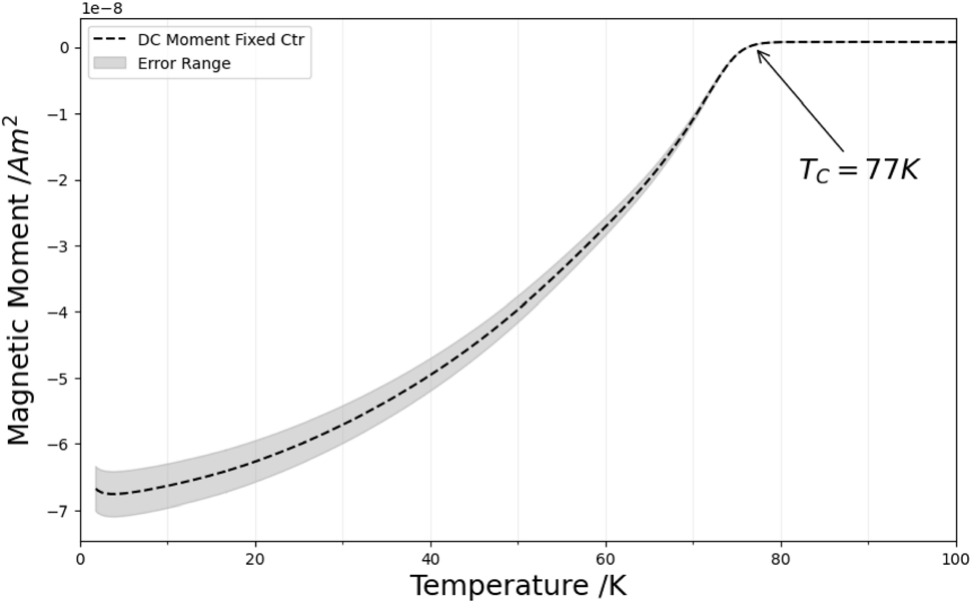
SQUID magnetometry measurement of BSCCO.

Rietveld refinement of the PXRD in Fig. S2† confirmed the presence of the expected BSCCO phases (the high-*T*_c_ 2212 phase and the lower-*T*_c_ 2201 phase) in the sample. SQUID magnetometry measurements in Fig. 11 showed a superconducting transition for the BSCCO sponge that is higher than the *T*_c_ of the calcium-deficient 2201 phase (8 K) but lower than that of the calcium-rich 2212 phase (77–92 K)^19–23^ The observed *T*_c_ (77 K) suggests a mixed-phase material and is consistent with other reports of BSCCO synthesized under non-ideal conditions. Its reduced *T*_c_ may be caused by not achieving the optimal oxidation state for the 2212 phase, as noted in prior studies.^23^

## 3 Conclusions

In summary, we have successfully used natural sea sponges (spongin) as a biotemplate for the synthesis of porous cuprate superconductors. The calcium present in the spongin fibers was readily incorporated into the ceramic structure, effectively doping YBCO and enabling the formation of BSCCO without any external calcium source. Electron microscopy confirmed that the sponge's intricate porous architecture was preserved in the superconducting materials, which could benefit cooling efficiency and reduce weight in practical applications. This biotemplating approach provides a greener and more sustainable alternative for superconductor fabrication, demonstrating how renewable biological materials can be harnessed in advanced inorganic synthesis. Future work should explore other classes of sponges (for example, calcium-rich *Calcarea*) and investigate how growth conditions or species differences affect their suitability as templates. Additionally, efforts to scale up the synthesis and optimize the superconducting phase (*e.g.*, improving the oxidation state for BSCCO 2212 to maximize *T*_c_) will be important for evaluating the feasibility of commercialization. Overall, this proof-of-concept study opens a novel pathway toward sustainable superconductor production, bridging materials science and biotechnology in alignment with global sustainability goals.

## Data availability

All underlying data are provided in full within this paper or in the ESI.†

## Author contributions

Conceptualization: JMU, SRH, JCS; methodology: JMU, JCS; investigation: JMU, JCS; visualization: JMU, SRH; funding acquisition: SJE; project administration: SRH, SJE, AJP; supervision: SRH, SJE AJP; writing – original draft: JMU, SRH, JCS; writing – review & editing: JMU, JCS, SRH, SJE, AJP.

## Conflicts of interest

There are no conflicts to declare.

## Supplementary Material

RA-015-D5RA00541H-s001
