# Supplementary material for: Morphological control of cuprate superconductors using sea sponges as templates
Source: RSC Adv. 2025 Apr 17;15(14):11189–93. doi: 10.1039/d5ra00541h (PMC12005474; doi:10.1039/d5ra00541h)
Supplement: RA-015-D5RA00541H-s001 [file RA-015-D5RA00541H-s001.pdf]

Supplementary Information (SI) for RSC Advances.
This journal is © The Royal Society of Chemistry 2025

Supplementary Information

1 Experimental

A YBCO precursor solution was prepared by dissolving metal salts described in Table S1 and BSCCO was prepared by using the ratio for calcium deficient precursor described in Table S2. A 2x2 cm piece of an unbleached sea sponge (supplier: Givereldi) was soaked in DI water overnight. After soaking, the water was squeezed out and the sponge was infused with approximately 2 ml of precursor solution. The infused sponge was calcined under the conditions described in Table S3.

Table S1 Composition of YBCO123 precursor solution, for 10 ml

Material	Amount /g
$Y(NO_3)_3 \cdot 6H_2O$	0.192
$Ba(NO_3)_3$	0.261
$Cu(NO_3)3 \cdot 2.5H_2O$	0.349

Table S2 Composition of BSCCO2201 precursor solution, for 10 ml

Material	Amount /g
$\mathrm{Bi(NO_3)_3 \cdot 5\ H_2O}$	0.485
$\mathrm{Sr(NO_3)_2}$	0.212
$\mathrm{Cu(NO_3)_2 \cdot 2.5\ H_2O}$	0.233
EDTA	0.500
880 ammonia	300 μL

Table S3 Calcination parameters for soaked sponges

Material	Temperature /°C	Ramp rate /°C min^{-1}	Dwell time /h
YBCO	920	5	4
BSCCO	830	5	4

SEM analysis was performed on a JEOL JSM-IT300 system. TEM analysis was performed on a JEOL JEM-2100 system. Powder X-ray Diffraction (PXRD) patterns were acquired on Bruker D8 Advance powder X-ray diffractometer equipped with a PSD LynxEye detector and utilising Cu-Kα radiation ($\lambda = 1.5418$ Å). Multiphase Rietveld refinement was performed in Profex 5.3.0.[1] Magnetometry analysis was performed using MPMS3-Evercool. The Inorganic Crystal Structure Database (ICSD) or Joint Committee on Powder Diffraction Standards (JCPDS) reference numbers for files used in the Rietveld refinement can be found in Table S4.

Table S4 Reference numbers for phases used in Rietveld refinement

Phase	Reference Number	Database
YBCO 0.98 1.98 2.91	65549[2]	ICSD
$\mathrm{BaCuO_2}$	1049[3]	ICSD
YBCO 211	72572[4]	ICSD
YBCO 143	65549[2]	ICSD
YBCO *Ca* doped	50098[5]	ICSD
CuO	04-007-137[6]	JCPDS
YBCO 123	62954[7]	ICSD
BSCCO 2212	203210[8]	ICSD
BSCCO 2201	65557[9]	ICSD

2 PXRD patterns

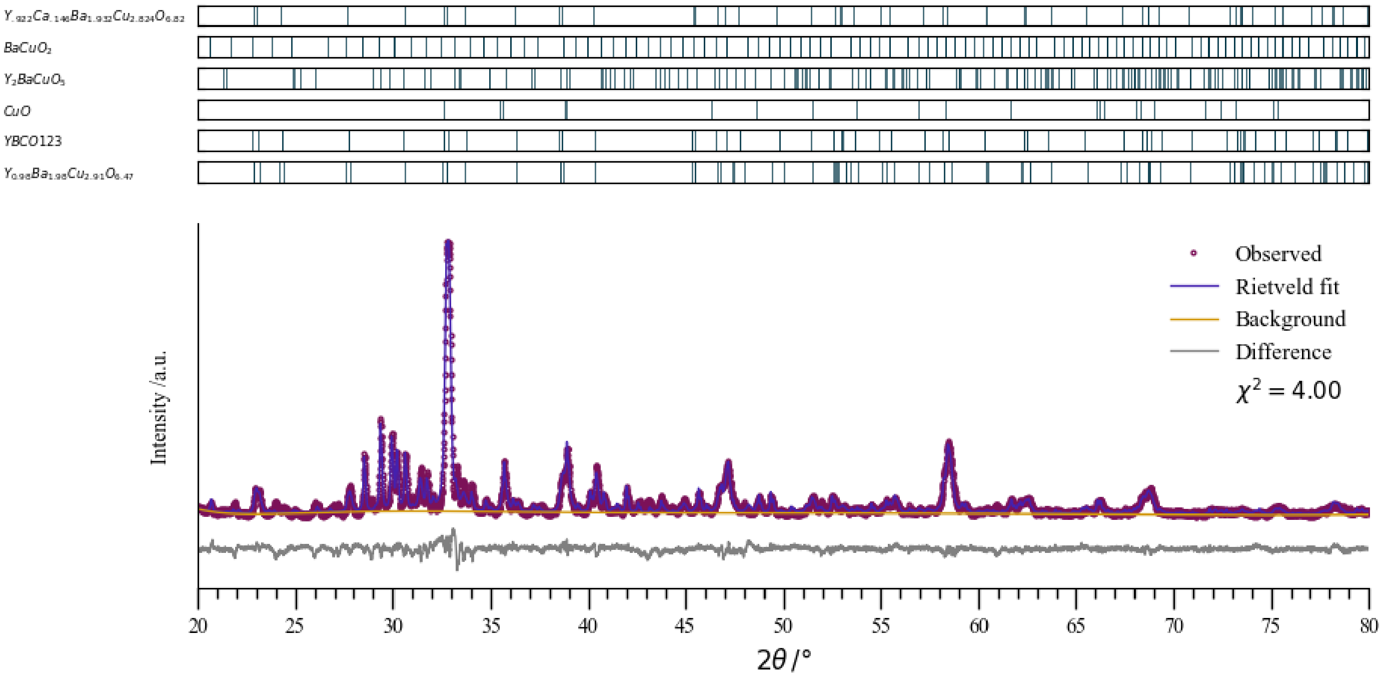

Figure S1 PXRD pattern for YBCO sample, composed of 33.4 % of calcium doped YBCO, 16.0 % of $BaCuO_2$, 15.9 % of YBCO 211, 13.1 % of CuO, 9.6 % of YBCO 123 8.2 % of YBCO 0.98 1.98 2.91, and 3.4 % of YBCO 143 (Not shown in the figure.). The composition was determined by Rietveld refinement analysis.

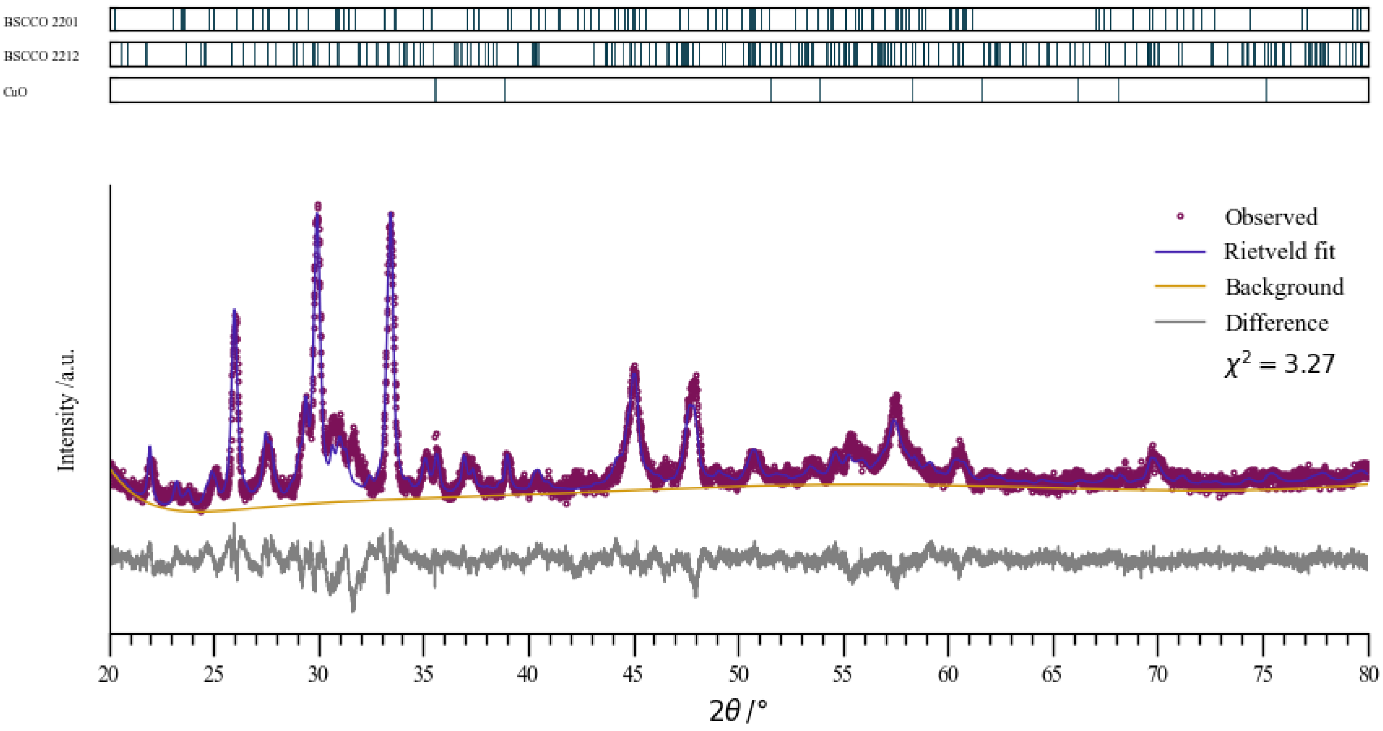

Figure S2 PXRD pattern for BSCCO sample, composed of 35.4 % of BSCCO2212, 56.2 % of BSCCO2201 and 8.4 % of CuO. The composition was determined by Rietveld refinement analysis.

Notes and references

[1] N. Doebelin and R. Kleeberg, *Journal of Applied Crystallography*, 2015, **48**, 1573–1580.

[2] F. Abbattista, M. Vallino, D. Mazza, B. M. Lucco and C. Brisi, *Materials Chemistry and Physics*, 1988, **20**, 191–199.

[3] R. Kipka and H. Müller-Buschbaum, *Zeitschrift für Naturforschung B*, 1977, **32**, 121–123.

[4] H. Shaked, H. Pinto, H. Ettedgui, Z. Gavra, M. Melamud, J. R. Johnson and J. J. Reilly, *Journal of Alloys and Compounds*, 1993, **194**, 13–17.

[5] G. Böttger, H. Schwer, E. Kaldis and K. Bente, *Physica C: Superconductivity*, 1997, **275**, 198–204.

[6] S. N. Kabekkodu, A. Dosen and T. N. Blanton, *Powder Diffraction*, 2024, **39**, 47–59.

[7] R. M. Hazen, L. W. Finger, R. J. Angel, C. T. Prewitt, N. L. Ross, H. K. Mao, C. G. Hadidiacos, P. H. Hor, R. L. Meng and C. W. Chu, *Physical Review B*, 1987, **35**, 7238–7241.

[8] R. E. Gladyshevskii and R. Flükiger, *Acta Crystallographica Section B: Structural Science*, 1996, **52**, 38–53.

[9] M. Onoda and M. Sato, *Solid State Communications*, 1988, **67**, 799–804.
